# Perceptions of government knowledge and control over contributions of aid organizations and INGOs to health in Nepal: a qualitative study

**DOI:** 10.1186/1744-8603-9-1

**Published:** 2013-01-18

**Authors:** Aditi Giri, Prashant Khatiwada, Bikram Shrestha, Radheshyam Khatri Chettri

**Affiliations:** 1 Kathmandu University School of Medical Sciences, Dhulikhel, Nepal

**Keywords:** Accra action agenda, Aid effectiveness, Coordination, International aid, Nepal, Paris declaration, Sector-wide approach

## Abstract

**Background:**

Almost 50% of the Nepali health budget is made up of international aid. International Non-Governmental Organizations working in the field of health are able to channel their funds directly to grass root level. During a 2010 conference, the Secretary of Population stated that the government has full knowledge and control over all funds and projects coming to Nepal. However, there are no documents to support this. The study aims to assess government and partner perceptions on whether Government of Nepal currently has full knowledge of contributions of international aid organizations and International Non-Governmental Organizations to health in Nepal and to assess if the government is able to control all foreign contributions to fit the objectives of Second Long Term Health Plan (1997–2017).

**Methods:**

A qualitative study was performed along with available literature review. Judgmental and snowball sampling led to 26 in depth interviews with key informants from the government, External Development Partners and International Non-Governmental Organizations. Results were triangulated based on source of data. Representatives of the Department of Health Services declined to be interviewed. Data collection was done until researchers felt data saturation had been reached with each group of key informants.

**Results:**

While Ministry of Health and Population leads the sector wide approach that aims to integrate all donor and International Non-Governmental Organization contributions to health and direct them to the government’s priority areas, questions were raised around its capacity to do so. Similarly, informants questioned the extent to which Social Welfare Council was able to control all International Non-Governmental Organizations contributions. Political tumult, corruption in the government, lack of human resources in the government, lack of coordination between government bodies, convoluted bureaucracy, and unreliability of donor and International Non-Governmental Organization contributions were identified as the main reasons for difficulties in aid integration.

**Conclusions:**

Despite its commitment to coordinate and control development assistance to the health sector, and its leadership position of the Sector Wide Approach, complete knowledge and effective coordination of all international contributions remains a challenge and is hampered by issues within the government as well as among External Development Partners and International Non-Governmental Organizations.

## Background

After a decade long civil war (1996 to 2006) and the abolition of monarchy, Nepal emerged as “Federal Democratic Republic of Nepal” in 2008 [1]. The Communist party of Nepal (Maoist) won the largest number of seats in the Constituent Assembly (CA) election in 2008 and formed a coalition government which included most of the parties in the CA [1]. For the first time in Nepal, the 2007 Interim Constitution declared that health is a basic human right and that the state bears responsibility for it. The Ministry of Health and Population (MoHP) is a governing body that aims at improving the health status of the population through “equitable and quality” health care services [2]. The current government led by the Maoist party is, however, over-burdened with issues of improving security and integrating the Maoists’ armies into the national army and may not be able to pay adequate attention to the health sector [3].

Jeremy Shiffman, in a 2007 article [4], conveys that to bring about a health priority in a country, two major factors are involved: first, significant political changes such as the introduction of democracy or decentralization that lead to modifications in the policymaking process and second, the priorities that are already present and will have to be competed against for available resources. He further states that to achieve health objectives, it is not sufficient to have donor support, resources, and effective medical and technical interventions. The objectives should also be a political priority [4]. Thus, politics of a nation shape its health objectives and is just as important as donor involvement and resource disbursement.

As a developing country, Nepal is heavily reliant on foreign aid for its development as well as for other social, economic and political initiatives. Aid agencies and their functionaries have been widespread in Nepal’s policymaking, legislative reforms and program design and implementation for over six decades [5,6]. A particular domain that is intrinsically related to and influenced by the aid regime is the country’s policy process. In the fiscal year 2010–2011, international aid represented 26% of national budget and 5.8% of Nepal’s Gross Domestic Product (GDP). The main sectors receiving external support are education, local development, roads and health. Nepal receives official development assistance (ODA) from over 40 donors, including 35 resident agencies. The Ministry of Finance (MoF) is responsible for the overall coordination of foreign aid. The Foreign Aid Coordination Division of the MoF is mandated to oversee the Government’s activities in the area of aid coordination, harmonization and alignment [7].

Foreign aid to Nepal is provided by Organization for Economic Co-operation and Development (OECD) donors, International Financial Institutions (IFIs), United Nations agencies, global vertical funds and providers of South-South cooperation. In fiscal year 2010–11, of the US$ 1.08 billion donated, approximately 58% came from multilateral donors, 36% from OECD bilateral donors and over 6% from bilateral South-South cooperation partners [8]. Of bilateral donors, India, China, Japan, Germany, United States, United Kingdom, Japan and Norway are the major ones. The largest multilateral donors are the World Bank Group, the Asian Development Bank, the United Nations Country Team, the European Union and the Global Fund to fight AIDS, Tuberculosis and Malaria [8].

Bilateral and multilateral agencies disburse ODA for distinct areas including, project support, Sector Wide Approach (SWAp), program support and humanitarian assistance. In 2010–11, they disbursed 63.1% for project support, 21.1% for sector wide development, 12.9% for program support and 2.9% for humanitarian assistance. However, the amount of disbursement was less than that of commitment. In 2010–11, of all government bodies, MoHP received aid from the highest number of development partners and got the largest amount of new foreign aid commitment, receiving 81 projects from 21 development partners [7]. The primary body under MoHP responsible for establishing relationships with External Development Partners (EDPs) and IFIs is the Department of Health Services (DoHS), with the objective of “enhancing effectiveness and developing health services and assist the MoHP in receiving foreign aid by clearly identifying the area of cooperation” [9]. Foreign contributions in the form of International Non Governmental Organizations and Non Governmental Organizations (I/NGOs) programs are overseen by the Social Welfare Council (SWC) [10], an autonomous government organization chaired by the minister of Women, Children and Social Welfare Ministry, and run by a board composed of members from several ministries including MoHP [10]. Within the MoHP, the Policy Planning and International Cooperation Division (PPICD) officially manages cooperation with national, international, I/NGO, and private sector stakeholders. The division falls under the responsibility of the Chief Public Health Administrator, who reports directly to the Secretary [11].

The MoHP of Nepal has a national plan that prioritizes horizontal health programs that cover the most important health needs of the Nepali people [12-14]. Contributions to the health sector come from a variety of sources including projects funded and often operated by international aid organizations and INGOs. While the government needs to approve these projects to assure that they fit the national plan,these organizations may have their own priorities, which may not necessarily fit within the ministry’s plans [15].

In 1953, the WHO Executive Board stated that any help to a country’s health status should be made by programs designed to strengthen its health care services and to address the most pressing health issues of majority of the population [15]. Health outcomes improve when primary health care is fortified [16-18]. The 2005 Paris Declaration of Aid Effectiveness places local government in the leading seat, calling for ownership, harmonisation, alignment, results and mutual accountability. Accra Agenda for Action, 2008 endorsed to ‘deepen implementation’ of Paris Declaration [19]. Evidence from other developing countries show that international funding in the field of health may not be in the best interest of the country’s existing health system [20-22]. Nepal government has laid out rules ensuring that government has full knowledge and control over all internationally funded and supported health related projects. Several publications by the MoHP give the impression that international donors and INGOs working in the field of health, work in close coordination with the ministry to fulfill its main agendas. The second long term health plan 1997–2017 talks about establishment of a coordinating body with access to information on health expenditure of all ministries, INGOs, NGOs and private sector [23]. It also states that all donors will be encouraged to consider the impact that their interventions have on areas other than the ones their programs support [22]. However, there is no government document to verify the extent to which this is applied.

MoHP leads the SWAp [23,24], bringing all stakeholders together, bringing reform in systems, structures and policies of MoHP in program financing and changes in relationship with other stakeholders within the sector including government agencies, multilateral and bilateral EDPs and the civil society (I/NGOs) [25,26]. However, as almost 50% of health spending comes from international aid, it is difficult for government to maintain clear leadership [5,6,27]. In a 2009 report on health aid effectiveness in Nepal, the author observes that although with SWAp, gains were made in reproductive health service delivery and child health, these successes cannot be attributed to international aid with certainty [27]. Another report on health aid effectiveness in Nepal states that contribution of I/NGOs at grassroots level is more significant than donor aid as I/NGOs are able to channel more of their funding to that level. By not channeling their funds through the government, they can avoid a large part of transaction costs [28,29].

Under these circumstances, this study aims to explore government and partner perceptions of the extent to which GoN currently is aware of contributions of EDPs and INGOs to health in Nepal and its control of foreign contributions in order to meet the objectives of Second Long Term Health Plan (1997–2017).

## Methods

A qualitative study was performed along with available literature review. For the qualitative study, taking in depth interviews (IDI) was the method undertaken. Judgmental sampling followed by snowball method led to 26 in depth interviews with key informants (KIs) from November 2010 to September 2011. Sample size was based on unique case selections made a priori and key informants were added to the list later through snowball method. Trustworthiness of KIs was insured by the fact that the researchers asked each organization to recommend people who may represent their organization’s points of view. They were people who had worked or were currently working with the government, EDPs or major INGOs for a minimum of five years and were involved in coordination efforts between the government and EDPs or INGOs. Formal and informal talks, telephone conversations, and office visits were made to track key informants’ opinions and experiences. All four researchers were mandatorily present at the main IDIs with each KI. Membership checking for qualitative data was done to increase validity of the data. The interview notes were reviewed with the key informant present who was asked if that was what he/she meant to convey.

Key government informants included the officials from MoF, Ministry of Women, Children and Social Welfare (MoWCSW), MoHP, National Planning Commission (NPC), and SWC. Key informants from EDPs were representatives from United Nations Population Fund (UNFPA), UN Children’s Fund (UNICEF), World Health Organization (WHO), United States Agency for International Development (USAID), Deutsche Gesellschaft für Internationale Zusammenarbeit (GiZ), Swiss Agency for Development and Cooperation (SDC), Kreditanstalt für Wiederaufbau (KfW), Australian Agency for International Development (AusAID), United Kingdom’s Department For International Development (DFID), and World Bank [28]. INGO key informants belonged to Action Aid, International Committee of the Red Cross (ICRC), United Mission to Nepal (UMN), Merlin, Adventist Development and Relief Agency (ADRA), Family Planning Association of Nepal (FPAN) and Family Health International (FHI).

One government organization that could not be represented in our study is the Department of Health Services. Although the research team repeatedly approached this organization from November 2010 to September 2011 and requested interviews with KIs belonging to it, appointment was not granted. Table 1 enlists all the organizations from which KIs were interviewed and also indicates the number of interviews from each organization.

**Table 1:** key informants, organizations and number of interviews from each organization

**SN**	**Organization**	**Number of interviews**
	**Governmental organizations**	
1.	Ministry of Health and Population	5
2.	Ministry of Finance	1
3.	National Planning Commission	1
4.	Ministry of Women, Children and Social Welfare	1
5.	Government’s zonal hospitals in two rural locations	2
Total number of interviews from Governmental organizations		10
	**External developmental partner organizations**	
1.	USAID	1
2.	AusAID	1
3.	GiZ	1
4.	KfW	1
5.	SDC	1
6.	WHO	1
7.	World Bank (the KI worked with World Bank in the past)	1
8.	DFID	1
9.	UNFPA	1
10.	UNICEF	1
Total number of interviews from EDP organizations		10
	**International non-governmental organizations**	
1.	ADRA	1
2.	Merlin	1
3.	ICRC	1
4.	FHI	1
5.	Action Aid	1
6.	UMN	1
Total number of interviews from INGOs		6

Notes from the IDI interviews were transcribed immediately by the study team and stored in the Principal investigator’s laptop. The taped conversation and its transcript were used to verify the transcription later. This data was analyzed using pile sorting and content analyzing. Data collection was done until the researchers felt that data saturation had been reached with each group of KIs- Government, EDPs and INGOs.

The broad themes and categories for the pile sorting were decided on by the researchers during two meetings where they discussed their impressions from the interviews. The categorization of data was done such as to understand how GoN interacts with and coordinates work with international parties. Impressions from IDIs indicated that the mechanisms for coordination with EDPs were separate from, and unrelated to, mechanisms for coordination with INGOs. Thus, we sought to understand them separately via themes I and II. It was also evident that compilation and integration of inputs from EDPs and INGOs by the government largely depended on how well government bodies were able to communicate with each other. Thus, theme III was chosen for this purpose. Theme IV was included to summarize the most frequently cited difficulties by all three parties as there was considerable overlap. Three items (shown in Table 2) were added to the preformed list. These additions were made by the researchers during the pile sorting phase after the need of adding them was seen (Table 2).

**Table 2:** Themes and categories used for pile sorting and data analysis

**Themes**	**Categories**	**Sub categories**
Theme I: Relationship between GoN and INGOs		
	A. Government Knowledge of INGOs working in the field of health	
	B. Coordination between INGOs and GoN	1. General agreement technicalities
		2. Project agreement technicalities
		3. Compliance of INGOs with SWC rules*
		4. Monitoring and Evaluation by SWC
		5. Competence of SWC
Theme II: Relationship between GoN and EDPs	A. Government Knowledge of work done by EDPs	
	B. SWAp	1. Support for SWAp*
		2. GoN as leader of SWAp
		3. Achievements due to SWAp
	C. Aid Flow	1. Aid flow through government channels
		2. Aid flow through non government channels
	D. Coordination Mechanisms between GoN and EDPs	
Theme III: Coordination within the GoN		
Theme IV: Difficulties Encountered by Government, EDPs and INGOs working in the Field of Health		
Theme V: Remarkable observations*		

*Three additional Themes and Sub-Categories were added later during the pile sorting.

Pile sorting was done in the following way:

• Each transcript was given a unique code. The total transcripts were divided equally among the four researchers. Each person did pile sorting of six transcripts, extracting points from them that fell under the broad themes and further into categories and subcategories.

• The transcripts were swapped among researchers and the process repeated. Thus, one transcript was summarized by two researchers.

• The summaries were compared by a third researcher who checked to make sure that both earlier people had categorized each point in the same manner. This researcher then made final summaries of the points that were agreed upon by both earlier researchers.

• Content analysis was done by the principle investigator and discussed with the whole group, including the consultant biostatistician.

Our literature review drew on the peer-reviewed scientific literature and relevant grey literature– reports, research and analyses, government reports, and web based materials. Initial searches used engines including PubMed and Google Scholar, using combinations of the search terms: ‘aid coordination’, ‘development assistance’, ‘aid’, ‘cooperation’, ‘aid effectiveness’, etc. from 2005. Our point of departure from 2005 is to include the progress made to fit the objectives of the Paris Declaration [27]. The study is approved by the Nepal Health Research Council (NHRC) (Ref. No. 1381) and follows all its rules.

## Results

### Relationship between GoN and EDPs

#### The sector wide approach

Nepal’s health SWAp was started in 2004, based on the Paris Declaration and Accra Agenda for Action. Members from EDPs, private sector, INGOs, NGOs with MoHP, NPC and MoF were involved right from policy making such that they knew what they would be supporting and would have a say in setting priorities. It was decided that all stakeholders would support the Second Long Term Health Plan (SLTHP) 1997 to 2017. The Health Sector Strategy was prepared based on SLTHP and the results of numerous studies that evaluated policy, service delivery, quality of services and system development. It outlines areas that need support in terms of complete support, technical support and parallel support. The Nepal Health Sector Program Implementation Plan (NHSP-IP) is used as a basis for channelizing donations. It dictates all functional policies of the government, with clearly defined goals that state the baseline statistics and targets to be achieved in the next five years and has a list of priorities termed, ‘essential health care package’. However, not all EDPs have signed up to support SWAp.

The statement of intent of SWAp states that all donors should support the GoN’s programs but they are not obliged to join the pool fund. All grants through pool fund are part of the government budget, making up around 17% of health budget. It is kept in Foreign Currency Account (FCA) in Nepal Rashtriya Bank (National Bank of Nepal) and spent by the GoN and EDPs in partnership. Reports on expenditure by the government are submitted to all EDPs every four months. Money that is not pooled is ‘direct fund’ and is not part of the government’s budget. Both pool funds and direct funds are mentioned in the red book and are to be spent according to government rules. However, some EDPs have agreements with the government on how to spend the direct funds and their money is ear marked for specific programs such that it is not under GoN control.

EDP KIs admitted that before the implementation of SWAp in 2004, their organizations supported vertical projects and now they support the entire health sector via SWAp. Some KIs said that sometimes their organization might be funding vertical programs, but they are all programs to support SWAp. While it is not mandatory for EDPs to pool their funds, a number of non pooling donors have signed a letter of intent promising to eventually pool their funds. This excludes the organizations such as the UN agencies and USAID whose internal structure does not allow them to do so. Some EDP KIs feel that the government is unable to control the non pool funds as these are “not bound to follow government rules and EDPs contract out projects (run by these funds) to INGOs”. No INGO KI stated that their work involved any support for SWAp. Some did, however, state that their work is all based on NHSP- IP and that it involved government health system strengthening. Although donors have a say when deciding on programs, government KIs said that the GoN makes final decisions independently and also decides on the spending, procurement and how monitoring and evaluation will be done. The GoN does not accept programs that do not go with government policies.

A little more than half the EDP KIs felt that SWAp is a good mechanism as, “donors and the government both have more accountability” and SWAp, “puts the government in the driving seat”, exemplified by a 2004 MoHP decision that instead of creating a separate HIV program, as suggested by an EDP, the program’s funds should be used in a more horizontal approach. One KI said, “The GoN has good control over the pool funds. It sets aside more than seventy percent of the health budget for essential health care as per NHSP”. Another said, “The MoHP has functioned well throughout the civil conflict and was also awarded international award for achieving MDG goal 5 in September 2010. Control by the GoN is seen mainly because of the powerful understanding and coordination between it and donors”.

A few EDP KIs feel that GoN is unable to implement programs on its own because of its shortcomings such as lack of technical expertise and resources for the broad field of work, a poor capacity to monitor and evaluate, and political conflicts that weaken the system. Government KIs agreed and said that the SWC and MoHP are both weak and require international financial and technical support. The programs that EDPs bring in are accepted by the government without question. One KI said, “Theoretically MoHP is the leader but in practicality EDPs are acting a lot like leaders”. Despite all this, one EDP KI said, “SWAp seems to be working in areas where the government is neither very strong nor very weak, like in Nepal. If the government is strong, we don’t need SWAp or development partners”. Similarly, an EDP KI conveyed that the transfer of power and intelligence from EDPs to GoN is a must and, “GoN is in the right track”. After SWAp was implemented, the proportion of foreign grants in the health budget has decreased from 55% to 50% and that the IMCI program is now entirely government driven. A government KI enumerated two achievements after SWAp implementation: the decrease in maternal mortality rate leading to Nepal achieving a MDG award in 2010 and the improved health seeking behaviour of the Nepali people, at the same time pointing out that the achievements cannot be entirely attributed to SWAp as it would require many studies to prove that.

#### Aid flow

Foreign monetary aid to the health sector in Nepal comes either in the form of grants/loans from EDPs that have an agreement with the government or via INGO run projects. EDPs may be bilateral or multilateral donors. Whereas earlier all money from donors came as grant, now only 40-50% is grant and the rest is loaned money. EDPs give grants to INGOs as well, but this is not seen in the red book. INGOs may or may not notify their grants to the MoF, but they always notify to SWC. Some EDPs give a percentage of their total grants not to the government but directly to INGOs that bring forth programs that fall into the sector approach as directed by the GoN. The money going to INGOs is not part of the government’s budget. However, INGOs’ accounting profiles are mentioned in the annex of the budget.

The red book has all the information about all funds from donors and also about how much grants particular donors have given for particular government programs. The pool fund money is part of the government budget but direct grants are shown in a separate column. The health ministry of Nepal Government receives grants in four ways:

1. General Budget Support (GBS)- The money is given to the MoF which then decides on where to spend it. In the opinion of one government KI, this is the ideal kind of support.

2. Sector Budget Support- This is in support of the SWAp. Four donors pool the money and the others don’t. Most of the sector budget support money is pooled- nearly 50%.

3. Direct earmarked money- This can either be spent through the government or the EDP can directly implement programs with this money.

4. Commodity grants- This is rare. Grants are not given as money but as commodities such as medication, vaccines, contraceptive products, etc.

Pool fund money is spent at the discretion of the GoN after discussion with pool donors. There are no problems in auditing as one report is sufficient. One government KI said, “The MoHP prefers pool donation because it gives more control to the government”. The non- pool fund grants have to be audited separately and this poses a problem as there are different auditing times for GoN and various EDPs due to differences in the fiscal year periods. Donors that ear mark their grants first talk to the MoHP and do their research before deciding on what areas to fund and then come to the MoF to give the funds. However, one government KI felt that the ear marked money can be used for programs that are already being implemented, leading to duplication.

Different EDPs give different percentage of their total grants to the government: one EDP only gives the government five percent of its total grants meant for Nepal while another one gives 80% to the government. The rest go to specific projects run by INGOs. When one EDP KI was asked why their organization’s grants are not pooled, the answer was that the GoN does not use the donated money effectively. Another KI argued, “I don’t think that the use of grants would be more effective if donors didn’t go through the government. Without supporting the system, there is no sustainable support to the country”.

At the local level, the district level government bodies have some budget allocated by the GoN and additionally have their own sources of funds. Sometimes, centrally allocated budgets for some health programs may be directed towards particular districts only. One EDP organization gives one third of its grants to the district development fund and the rest to the central government. After the ‘local safe governance act’ was established in 1991 by the ministry of local development, local bodies have become self governing and the grants that go to them do not have to be notified to the central government. When asked why this EDP does not give grants to the central government, the KI replied that the government is not transparent and resources given to the central government are not channelled out to the local bodies in a timely manner.

Similarly, another EDP implements projects at the district level and does not contribute at all to the red book. The KI from this EDP feels that when giving money at the central level, it first has to go to the DHO, then to the local level government employee who ultimately tells a volunteer what to do and the volunteer does it for free. The money is used up from the central level down to the local level for works such as trainings, training of trainers (ToT) and master ToTs. Thus, this EDP prefers to go directly to the local level and invest there. The UN agencies and one other EDP provide technical support and rarely grant money.

#### Coordination mechanisms

One government KI summarized the GoN-EDP coordination by saying, “EDP is in good relation with the GoN”. Government and EDP KIs said that coordination starts at the policy making level. EDPs were involved from the early stages of formulation of NHSP I and II, such that they would know what they would be supporting and have a say in setting priorities. All government programs are implemented in collaboration with EDPs, INGOs and NGOs. Similarly, donors who give money for particular programs first consult with the government and then decide on what to support. For example, when it came to promoting home deliveries by SBAs, donors argued that this would be counterproductive because the international community supports hospital deliveries. The MoHP had to explain that for people in remote, rural areas where accessibility and cultural beliefs pose problems, the best solution is to send trained personnel to them. Donors then decided to fund this program instead of promoting hospital deliveries.

Coordination mechanisms in the Nepali health sector are comprised of several meetings held yearly or several times a year. Most important are the Joint Annual Review or JAR meetings that bring together all stake holders in the field of health. In the meetings, the government prepares about ten reports and presents on overall management, achievements and shortcomings of the work done in one year. GoN and EDPs evaluate the performance and productivity of the work done in the past year and plan future strategies. For effective coordination, INGOs, the civil society and media are also involved as a ‘check and balance’ mechanism. Each meeting lasts for two to three days. One KI said, “JAR meetings are good. There is bilateral discussion”. One EDP KI opined that the JAR meetings are effective, as both the EDPs and government sides critically analyze the reports and although these meetings may seem ‘formal and ritualistic’, they ultimately foster efficiency. Most EDP KIs thought that JAR meetings are not effective, “It (JAR) is more of a business transaction where people are not the centre and money is. It is ritualistic and no decision is made properly”. Another said that the results from the meetings are not all implemented. A third EDP KI said that people do not come prepared to the meeting and only complain, they fail to discuss many important issues and there are always differences in opinion between the government and the EDPs.

Other coordination meetings include the Joint Consultative Meeting or JCM, Joint Technical Assistance Arrangement or JTAA and Annual Work Plan Budgeting Meeting (AWPB). JCM are held every three months and achievements, problems and solutions are discussed. JTAA is a yearly meeting and the AWPB meeting involves the government, EDPs and civil society. The Joint Financial Agreement (JFA) brings together EDPs that pool their grants and those that give grants directly to the government. It outlines exactly how many meetings should be held in a year, what the agendas should be, etc. Reporting for pool funders and non pool funders is done in a joint manner under the JFA. All donors, except the UN agencies, accept a single government audit done by the office of the auditor general. This office reports on a four monthly basis. Furthermore, government and EDP KIs said that IHP + has helped cultivate a deeper coordination between GoN and EDPs.

The coordination among EDPs is good, as conveyed by most EDP KIs. The EDP forum meets as and when needed. However, one KI said that coordination of the EDP forum with India, China and Korea is not good as they are not part of the forum. Another KI said, “Sometimes the government wants to play off two donors so we try to team together and prevent this”.

In contrast to this, there were no comments by government KIs that implied that coordination within the government is good, whereas five government KIs stated that the coordination between and within government bodies is bad. “There is absolute lack of coordination and communication between ministries, among personnel of the same ministry and among different departments. People tend to hide information in order to implement programs they favour,” said one government KI. The presence of two secretaries at MoHP has posed some problems. Misunderstandings between the two and their frequently interchanging designations as secretary of health and secretary of population causes problems to donors.

The lack of communication between SWC and MoHP may lead to duplication of health programs. The SWC does not work under the MoHP and KIs from MoHP in turn feel that they do not need to know about INGOs and NGOs working for health. The MoHP is not informed about the evaluation of INGOs. INGO KIs told us that a recent conflict between SWC and other ministries as well as internal conflicts between government bodies had led to massive delays in the document processing process for INGOs. There is slow communication between ministries as well as between central and local level government bodies.

### Relationship between GoN and INGOs

#### Government knowledge of INGOs working in the field of health

KIs from the government all agreed that for any organization to work in the capacity of an INGO, it has to be registered at the SWC. Thus, SWC has full knowledge of all registered INGOs and the work they do. This is not true for other government organizations. The KI from MoHP stated, “MoHP has no knowledge of how many INGOs and NGOs are working in Nepal in the field of health and what work they are doing”. The MoF also only deals with donor organizations. Its reports do not reflect on the contribution of INGOs.

Although SWC is aware of all registered INGOS, government and INGO KIs stated that there are organizations that opt to “bypass the SWC” by not registering. One KI speculated that these INGOs do not deal at all with the government so as to avoid all the bureaucratic hurdles. Another said, “All INGOs that have an intention of actually working and doing some good will invariably come through the SWC. The ones that don’t are obviously fraudulent”. The KIs told us that they did not have any knowledge of such INGOs, although it is certain that they exist. The proof of their existence, however, is seemingly impossible to find. When asked if we could obtain some information on these “illegal” INGOs, these KIs told us that they did not know how.

Another issue that came up was of INGOs that are working openly and are exempt from the legal obligation of coming through SWC. All KIs from INGOs said that they either work with governmental organizations or in the very least inform the government about their projects in health. Of the seven INGOs we interviewed, four said that they were registered at the SWC while three said that they weren’t. Those that weren’t registered said that they all had special agreements with the government that made them exceptions to the one door policy. One of these INGOs is working in Nepal under the Geneva Convention and is a “neutral and independent body” that does not collaborate with the GoN or SWC. Another one said that their major donor has a “special agreement” with the GoN so that it does not have to go through the SWC, except for their Tuberculosis program which is registered at the SWC. The last one was conflicted upon what its status is- NGO, INGO or a hybrid organization and it too works in direct coordination with the NPC.

There are other organizations that work in the field of health but are not registered as INGOs but as companies, following an entirely different set of rules and having no obligation of reporting their work to SWC. When asked if SWC had any powers to take action against INGOs that refuse to get registered, the KI informed us that organizations legally had an option of not going through SWC and there was nothing SWC could do to make them comply with its rules.

#### Compliance of INGOs to SWC rules

Government KIs agreed that most INGOs comply with the government’s rules, including the 80:20 rule of money allocation, the preliminary need assessment, having a partner NGO and getting permission from local authorities. Before starting work in Nepal, INGOs have to sign two agreements- general and project. The general or blanket agreement states that no INGO can work independently in Nepal and must work with a ‘partner NGO’ and with SWC. Thus, there is a tripartite agreement between SWC, NGO and INGO.

The signed agreement is sent to the MoWCSW. Here, a facilitating committee reviews the papers. This committee has 10 representatives from various ministries: Ministry of Home Affairs, Ministry of Law and Justice, MoF, NPC, Ministry of Foreign Affairs, and in case of health related INGOs, MoHP. The committee recommends whether the INGO should be allowed to work in their specified field and geographical area. The MoWCSW takes this into consideration and either approves or disproves. The SWC then executes its decision.

Within three months of signing of the general agreement, a technical or project agreement must be signed. Before thinking of any project, the INGO first reviews all the plans and policies of the government like the SLTHP and also looks at various indicators. They use data from government surveys and also from studies done by UN agencies. Then, based on that and the wants of the donors, a project idea is made. They take this to the government and ask them where this particular project may be needed. The government suggests a district and the INGO goes there to do a need assessment. At the local level, it meets with the district development committee, or district health office - governmental bodies, and asks for permission to work in the area. The written permission is submitted with the project proposal to the SWC. This way, duplication of work at the community level is prevented. More often than not, the priorities of the donors are the same as that of the government. The donors themselves also consult with the government of the recipient country to make their country strategy. When the unlikely scenario of donor priority not matching with the government priority occurs, the INGO convinces the donor to follow government priority.

All approved INGOs bring their project proposals to the SWC, which checks if all requirements are fulfilled and then sends it to the project facilitating committee for approval. At the facilitating committee, the members base their judgement on whether the project fulfils any of the current fiscal year’s plans made by the government. The 80:20 rule is followed where 80% is the budget allocated to the project cost and 20% is for administrative functions of the INGO. However, a government KI informed us that there are various loop holes to this policy. For example, the field office fee and salaries can be included in the project implementation budget, although they may be administrative costs.

When asked how long this whole process takes, the KI from SWC told us that it takes from two weeks to a maximum of one month and that there have been delays in the past due to political conflict involving the facilitating committee. In some cases, some INGOs have been allowed to work after a “verbal agreement”- this work is limited to the preliminary setting up of equipment and hiring of man power. All INGO KIs agreed that the actual time taken is about five to six months minimum. They also admitted to starting projects without acquiring an official approval from the SWC. One KI expressed opinions on the project approval process, “When deciding on a project, we rarely consult with SWC and we do not wait for approval before stating a project. The approval takes at least six months and may take up to two years. Although according to the rules, INGOs are only supposed to implement after receiving approval, this is impossible and all INGOs know this. No donor will wait that long. Donors would understand if there were definite rules for the time period needed for the approval process. But, there is no such thing. Our INGO has hired a particular person full time to follow the project approval process at SWC. Another problem is, all personnel get changed when there is change of government and they take a long time to learn how to do their jobs”.

#### Monitoring and evaluation by SWC and competence of SWC

INGO KIs informed us that the monitoring and evaluation done by SWC is one of the three M&E done for INGOs. The other two are: one by the donor agency and one by the central headquarter of the INGO. The present SWC monitoring and evaluation system has only been implemented since 2009. All INGOs work on a project basis. They submit six monthly reports to the District Project Advisory Committee (DPAC) which is composed of members of the target population and representatives from the district governmental office. The DPAC gives feedback about the INGO’s work to the Central Project Advisory Committee (CPAC). CPAC holds annual meetings for individual INGOs. Depending on the reports of the DPAC and CPAC, the INGO may or may not continue its projects.

Furthermore, a final evaluation is done by a Nepali expert team that is composed of an experienced veteran of the field, a ministry representative, a member of the SWC and a financial expert. The team looks over the INGO’s documents and also goes for field visits and interviews the target population. The report submitted by this team undergoes a primary discussion at the SWC which then gives a verdict, stating if the project was effective in contributing to fulfilling national agendas or not and makes recommendations about accepting such projects in the future. The reports of the final evaluation are available freely at the SWC website: http://www.swc.org.np.

The process outlined above is applicable to all INGOs that are registered at the SWC. However, the KI from SWC told us that SWC has no role in monitoring INGOs that are not registered. Another KI stated that the process of acquiring preliminary permission from the DDC is an important step to prevent duplication. The DDC coordinates with other stakeholders already present in the area. However, their database of all the INGOs working in the area is deficient. When projects are implemented without prior government knowledge, sometimes duplication of work is created, as seen in the Village Development Committee (VDC) we visited. Despite the presence of a government run health post in this VDC most people visit a health center funded by international donors. While it has a full time staff, a well-stocked pharmacy and diagnostic equipment, its government counterpart is continuously understaffed and disorganized. Locals say they prefer the INGO health center over the government health post. The government health post doctor is frequently absent for long periods of time. The doctor was not available for interview when we visited the health post as he was on leave in Kathmandu and had been there for the past month. The attendant at the health post explained that the doctor runs a private clinic in the capital.

In line with this, we interviewed two doctors working at the Government’s Zonal Hospitals in two different rural locations. They both agreed that their official leaves last longer than the sanctioned time. Most of their leaves are to attend training programs held in the capital funded by international organizations. For a week long training program, they say they leave station for three weeks or longer, depending on transportation facilities available. The trainings are largely based on particular skill based practices or research practices and the doctors both agree that they do not gain much usable information from them. The incentive for them is mainly a paid trip to Kathmandu and some added money, usually amounting to more than their monthly salary. These incidences are issues of national interest and are frequently covered by the media. When further asked whether the doctors would work for the government or an INGO in their locality, if given the choice, they both said that they’d work for the INGO run service as it would provide more facilities and vastly better salaries.

One Government KI recognized that the SWC is under staffed and it should be decentralized to be a more effective governing body. Currently, there is only one office in the capital and there are plans to create offices outside. There are no definite fields of works such as health, environment, education, etc. Currently, the SWC is limited to looking after how much money an INGO brings in, how much it spends and what its logical framework is.

INGO KIs said that the monitoring visits are requested by the INGO itself and it does not occur often, further stating, “They (SWC) are such a small organization and it’s impossible for them to monitor every project by every INGO”. However, INGO KIs observed that unlike other areas such as education, the government is very much involved in health related projects. It appoints central person for different areas such as reproductive health who makes regular visits to the project site. At district level, the one KI informed us, the INGO works closely with the authority there and they are constantly monitoring it. The same KI also opined that the annual CPAC meetings are not worthwhile and that its impact and reason it is done are very vague.

One INGO KI said, “(We) do not go through SWC. There is nothing to prove by going through it. SWC has not been able to achieve anything”; that the audit done by its donors is “far stronger” than that done by SWC and that international humanitarian ethics are better to follow than SWC’s rules. Two other INGO KIs felt that the SWC M&E process is weak because of limited human resources. One of them also said that internal conflict and the tumultuous political situation are to blame for SWC’s shortcomings. In the same line, one EDP KI gave us an opinion on the SWC M&E process: “SWC monitoring is very weak. INGOs’ and NGOs’ health project output monitoring is non-existent. There is no mainstream database of NGO/INGO health related project audit.” Government KI from SWC also said that there were no project audits available to the public due to a lack of human resources at SWC.

### Difficulties encountered by government, EDPs and INGOs working in the field of health

All KIs were asked if there were any difficulties encountered by them or their organizations when working in the field of health in Nepal. There were 13 difficulties delineated that are categorized and presented in Table 3. The table also mentions numbers of KIs from the different categories who stated the various problems. The numbers are not meant to imply statistical significance but are given to clarify which groups of informants tend to hold which opinion.

**Table 3:** Problems stated by the Government, EDPs and INGOs for Aid Integration (N = 26)

**Difficulties**		GoN*	EDPs*	INGOs*	Total*	**Comments**
**Issues related to the political context and government stability, structure and capacity**						
1	Political instability	1	7	2	10	The change in Nepal’s ministers occurs frequently, bringing changes in personnel and policies in the MoHP, and influencing the way resources are handled. Personnel who are appointed politically often are not experienced or qualified. Political instability also brings frequent mass strikes and lack of security.
2	Lack of human resources in the Government	3	4	2	9	Despite the increase in budget allocation to health, increase in funds and programs, the MoHP still follows the personnel organogram created in 1993. The Policy, Planning and International Cooperation Division (PPICD) director’s post stood vacant for more than six months after resignation of the former director. The SWC does not have separate departments for health related INGOs and NGOs and there are too many INGOs for GoN to handle. One government KI said, “Weak government system in our country does not have the capacity to run enormous resources provided by international donors.”
3	Convoluted Bureaucracy	-	5	4	9	When working with government, processes are “painfully slow”. The SWC takes several months to several years to approve projects, a process which can only be fastened via bribes or personal connections. The money almost finishes before the project reaches the community due to numerous payments made to government officials on the way.
6	There are two secretaries in Ministry of Health and Population	1	4	-	5	The secretary of health and population’s roles are interchanged from time to time, making donors “annoyed” and confused. Their roles often overlap and are not clearly delineated creating “a sense of malaise” and one KI stated, “The two secretaries compete about going to particular meetings, for example the JAR meeting of January 2011, creating unnecessary complications.
7	Corruption in the government	-	3	2	5	Two EDP KIs said that their organizations used to give grants to the central government but stopped doing so and instead started giving grants directly to NGOs and INGOs.
11	Government’s system of monitoring and evaluation isnot effective	-	3	-	3	Government’s mechanisms of monitoring like JAR are not effective, people do not come prepared to them and a lot of issues are missed out.
13	Government does not use donated money effectively	-	2	-	2	In the early months of 2011, a large amount of grant money was stopped because the government’s financial report was not satisfying.
**Issues related to intersectoral relationships and alignment within the sector**						
4	Lack of coordination between government bodies	3	3	1	7	The news of new agreements between the government and EDPs or INGOs is not communicated between government bodies involved. There are internal conflicts between bureaucrats and technocrats in the MoHP. Due to lack of coordination between MoHP and SWC, there is a duplication of projects.
5	EDPs sometimes do not follow through with commitments	3		3	6	Donors and EDPs can be “unpredictable” in that they will promise a certain amount as grant, which is then added to the budget but later they do not follow through. Some donors continue to support more vertical programs that do not support SWAp, working under agreements that were signed prior to implementation of SWAp.
8	Absence of India and China in the EDP forum	-	3	-	3	Both India and China contribute to Nepali health system in their “own piece meal approach”, making hospitals, donating ambulances, etc. without consulting any other EDPs. They don’t participate in coordinating meetings.
10	Problems in auditing due to differences in fiscal years among donors and Nepal Government	1	2	-	3	
**Issues related to competence of government employees**						
9	Difficulties working with local level government bodies	1	1	1	3	There is a lack of ownership in the district level, as government officials expect the EDP or INGO to take full responsibility of any projects they fund. The DDC is supposed to coordinate all stakeholders working in the community, but they don’t have complete database of all organizations working in the area.
12	Non professionalism of government employees	-	2	-	2	GoN personnel do not attend meetings regularly and sometimes postpone them for personal reasons. The GoN does not take the initiative to organize meetings and has problems with keeping deadlines.

* These numbers are not meant to imply statistical significance but are given to clarify which groups of informants tend to hold which opinion.

## Discussion

For an agriculture-based, undeveloped economy like Nepal, progress efforts have been largely driven by foreign aid and INGO funded work. Nepal’s budget for 2011/12 shows that over a quarter of government revenues were provided by foreign aid and loans [30]. Over the period of 2004 to 2009, international aid have made up 40% to 50% of the health budget. This dependency carries risks at a time when concern of corruption within government of Nepal can play into the hands of international donor fatigue [28,31,32].

All EDPs that support the Nepali health system support SWAp. They either directly fund the government’s programs or fund INGO programs that support SWAp. This study did not find any instances where a donor only supported vertical programs. This is similar to the finding of a 2010 systematic review of integration of targeted health interventions into health systems. The authors observed that no interventions were fully vertical or horizontal and instead the picture is ‘highly heterogeneous’ [33].

SWAp in health was started in 2004 in Nepal. Donors as well as recipients prefer this approach to the earlier project approach. It is supposed to reduce duplication, lower transaction costs, improve aid effectiveness, and strengthen national leadership and health system [34]. In our study, EDP KIs admitted that before 2004, vertical projects were being run and that after SWAp, the whole health sector is supported through a horizontal approach. Priorities are set according to national need. The guiding document is the NHSP-IP, which was formulated under the leadership of the ministry of health and population with inputs from EDPs and the civil society.

EDP and government KIs believe that SWAp is working well in Nepal. They credit SWAp for the fact that the health sector performed well even during the years of internal conflict (1996–2006), and also for Nepal winning the Millennium Development Goal (MDG) award for achieving goal five in 2010 [35]. They also say that SWAp has helped reduce duplication of work and has fostered a better relationship between GoN and EDPs. This is further supported by a 2009 World Bank report that states that with SWAp, achievements were made in Nepal in reproductive health service delivery, child health, and family planning and health system strengthening [27].

On the other hand, KIs also agreed that SWAp’s actual impact can only be assessed after about 10 years of sustained implementation [34]. Similarly, credit for the achievement of MDG goal five also cannot be given to SWAp or to any one donor as health resource tracking in a system where donors contribute to pool funds and give budgetary support is very difficult. When funds are disbursed through the government, they can be allocated freely and interchangeably to various programs making it difficult to attribute them to specific areas [36].

The leadership role in SWAp is given to MoHP [13]. It should make sure that any intervention in health is complementary to and not a replacement of the existing systems [37]. The opinions of KIs in our study were almost equally divided in this respect with some saying that GoN is a good leader, some saying that it is a bad leader and others saying that it is growing better as a leader. Evidence from other countries show that the leadership role can be ‘problematic’ because of limited leadership capacity as seen in Rwanda, poor relationship with the ministry of finance as seen in Mozambique, and change of senior management as in Zambia [27]. We found the MoHP’s leadership capacity is poor due to the unnecessary presence of two secretaries, poor coordination with other ministries and the SWC and deficient communication between personnel inside the ministry. The frequent changes of personnel due to political instability cause problems with inter- organizational relationships, the transference of information and institutional memory.

Although the norm in a SWAp is for donors to pool funds [27], in Nepal donors are not obliged to do so. About 17% of health budget is pooled grant money. Grants can also be given directly and not pooled. This is termed ‘direct fund’ and does not form part of the government’s budget. This money is spent after a special agreement between GoN and the donor. It is earmarked for specific programs. One EDP KI said that their organization’s money is not pooled because the GoN does not use the money effectively.

A 2009 article on health aid flow over the last 10 years discusses that even though budget support and sector support are some of the most effective forms of aid, the proportion of aid that goes through these channels is low [38]. We saw that EDPs give various proportions of their grants to the government, spending the rest on projects run by INGOs. One major donor only gives 5% of its grants allocated for Nepal, to the government. Money going to INGOs is not part of the government’s budget but the INGO’s accounting profiles are mentioned in the annex of the budget. Another donor gives one third of its grants directly to the district because it feels that government is not transparent and money given at the central level is not channelled out to the local bodies in a timely manner. In the 2010 Corruption Perception Index score by Transparency International, Nepal is ranked 146th out of 178 countries with a score of 2.2 [39]. On a more positive note, a 2009 study on health aid effectiveness in Nepal states that INGOs are able to spend more of their funds at the community level so that their contribution at that level is more significant than aid provided by donors [28]. However, without proper regulation and reporting, there are doubts as to whether INGO contribution actually helps fulfil national goals.

Government accountability is established when there are pressures from the civil society and service beneficiaries, political pressures within itself, and an independent audit office support [36]. In Nepal, all donors except the UN agencies accept a single government audit done by the office of the auditor general that reports on a four monthly basis. All donors, be it the ones that pool or don’t pool their grants, have signed the JFA whereby reporting for pool funders and non pool funders is done in a joint manner and coordination meetings are held regularly. There is also the involvement of civil society in coordination meetings such as JAR. These facts indicate that there is government accountability in Nepal. However, in our study, most KIs said that JAR meetings are not effective. These meetings have become ritualistic, yield no coherent decisions and decisions that are made are not implemented. People do not come prepared and many issues are missed out. JAR meetings are important tools to assess the sector’s achievements and shortcomings. However, their success depends on the expertise and experiences of the people involved [28].

For any governing body to function optimally, it must have full knowledge and control of internal and external factors working in its realm. Our government KIs told us that INGOs coordinate with the SWC at the central governmental level. Other government bodies such as the MoHP and MoF do not get involved with issues of INGOs and do not have any knowledge of their work. There is no effective communication between SWC and other government organizations. The MoHP and MoF only deal with donor organizations and their reports do not reflect contributions of INGOs. KIs from all three categories stated that SWC is not competent to handle all INGO because it is understaffed and does not have separate departments to manage INGOs from different sectors. From our interview with the representative from SWC, we learned that personnel there do not know anything about SWAp, and had never heard it mentioned before. SWC is the organization that approves or disproves programs proposed by INGOs. In this context, there is no mechanism to ensure that INGO programs also contribute to SWAp.

Several KIs revealed that the SWC has knowledge only of INGOs that have been registered there. The latest list (Sept 2011) of registered INGOs available at the SWC website shows that of the 199 INGOs registered, 67 work in the field of health [13]. There are organizations that work in the capacity of an INGO in health but are registered elsewhere and follow entirely different sets of rules, having no obligation of reporting their work to SWC. One such organization is registered as a company. The SWC does not have any power to direct these organizations to come through the proper channel. During the course of our study, we came across several INGOs that are currently working in Nepal for health and are not registered at the SWC. KIs from these organizations explained that although they may not work with SWC, they work with other government bodies and keep the government informed of their work.

Both government and INGO sources agreed that INGOs adhere to most rules set by the SWC such as the 80:20 money allocation rule, they all work with local NGOs and community level government bodies. However, dialogues with KIs working at rural government hospitals suggested that at the local level, INGO clinics duplicate work done by government facilities and are diverting skilled health workers from government jobs to better paying INGO jobs. Trainings paid for by donors and INGOs deprive government facilities of health workers for long periods of time. Although all sign the general agreement and prepare project agreements, they generally do not wait for SWC approval before starting projects, as stated by KIs from government as well as INGOs. INGO KIs explained that this was due to long delays in the process. These projects, thus, may not fall within government agenda. INGO KIs, however, explained that they do a full needs-assessment before designing any project to make sure it fits within government plans.

When we categorically asked our KIs about any difficulties they faced in their work in the health sector, most government KIs said that everything was going according to plan. Most difficulties were faced by EDP and INGO KIs, as described in Table 3 and most dealt with shortcomings on the government’s side. This included issues of political instability, government instability, structure, capacity and alignment within government sectors, and competence of government employees. The government KIs who stated problems also stated those dealing with government short comings including political instability, lack of human resources in government, two secretaries in MoHP, lack of coordination between government bodies and difficulties working with local level government bodies.

## Conclusion

This study reflects the perceptions of key people in the government, EDP organizations and INGOs who have worked in the field of public health in Nepal for at least five years. Through it, several mechanisms of the government’s handling of international support and the problems involved have come into light. The health system SWAp is implemented in Nepal with the objective of better aligning government agendas with contributions of foreign organizations to health. By definition, it puts the MoHP in the driving seat. Under SWAp, the MoHP is thought to have complete knowledge of and control over foreign contribution to health which is comprised of funds from EDPs and international donor funded INGO projects. Large proportions of funds from EDPs also go to INGO run projects. I/NGOs are largely overseen by a separate government organization, the SWC, which does not directly work under MoHP.

Through our study, we saw that although the system of SWAp is in action, the MoHP has very little knowledge of contributions of INGOs to health. The government body that regulates INGOs, SWC, is not aware of what SWAp is and is unable to say whether it has a complete database of all INGOs working in the field of health. There are INGOs that do not get registered at SWC and there are also those that do not wait for SWC approval before implementing their projects. Government knowledge of their work is deficient.

We also saw in our study that MoHP works well with EDPs. However, EDPs do not always only give grants to the government. They also fund INGO run projects and not all EDPs pool their grants. The non- pool grant money is not completely under MoHP control.

The strength of a government as the leader is reflected in how it is run. The MoHP is still following an 18 year old organogram of hierarchy. It also redundantly has two secretaries who do not communicate well between themselves. The personnel in MoHP and other ministries are changed frequently, with every change in government causing problems in relations with EDPs and in the smooth running of day to day work. Lastly, a governing body in control must have a good communication and coordination system within itself. This was found to be deficient in GoN. Not only was there little communication between ministries, the communication between personnel from the same ministry was also poor, exemplified by the disconnect between the two government organizations that are most involved in handling international contributions to health: MoHP and SWC. Another government organization involved extensively with coordination with EDPs, the Department of Health Services, declined interview and the research team was unable to include their points of view. Nonetheless, the health sector in Nepal did well even during the years of conflict and majority of our KIs said that the MoHP is doing a good job as the leader of SWAp and is also growing stronger as a leader.

## Abbreviations

ADRA: Adventist Development and Relief Agency; AusAID: Australian Agency for International Development; CPAC: Central Project Advisory Committee; DFID: Department for International Development; DPAC: District Project Advisory Committee; EDP: External Development Partner; FHI: Family Health International; FPAN: Family Planning Association of Nepal; GiZ: Deutsche Gesellschaft für Internationale Zusammenarbeit; ICRC: International Committee of the Red Cross; INGO: International Non Governmental Organization; JAR: Joint Annual Review; KfW: Kreditanstalt für Wiederaufbau; KI: Key informant; MDG: Millennium Development Goal; MoF: Ministry of Finance; MoHP: Ministry of Health and Population; MoWCSW: Ministry of Women Children and Social Welfare; NHRC: Nepal Health Research Council; NHSP-IP: Nepal Health Sector Program Implementation Plan; NPC: National Planning Commission; PPICD: Policy Planning and International Cooperation Division; SDC: Swiss Agency for Development and Cooperation; SWAp: Sector Wide Approach; SWC: Social Welfare Council; UMN: United Mission to Nepal; UNFPA: United Nations Population Fund; UNICEF: UN Children’s Fund; USAID: United States Agency for International Development; VDC: Village Development Committee; WHO: World Health Organization.

## Competing interests

The authors declare that they have no competing interests.

## Authors’ contributions

AG was the principle investigator, was involved with conceptualization and study design, literature review, creating connections with key informants, conduction of interviews, data analysis, manuscript drafting. PK was also involved in conceptualization and study design, literature review, conduction of interviews, data analysis and helped with manuscript drafting. BS helped with study design, literature review, and conduction of interviews. RKC assisted in conducting interviews. All authors read and approved the final manuscript.
